# Comparative Assessment of the IR Biotyper and Pulsed-Field Gel Electrophoresis (PFGE) for Epidemiological Surveillance of *Klebsiella pneumoniae* in an Oncology Hospital

**DOI:** 10.3390/jcm15062301

**Published:** 2026-03-17

**Authors:** Maria Szymankiewicz, Karolina Węgrzyńska, Anna Szczepańska, Lidia Baraniak, Anna Wawrzyk, Anna Baraniak

**Affiliations:** 1 Department of Microbiology, Prof. F. Łukaszczyk Oncology Centre, 85-796 Bydgoszcz, Poland; szczepanskaa@co.bydgoszcz.pl; 2 Department of Pharmaceutical Microbiology and Laboratory Diagnostics, National Medicines Institute, 00-725 Warsaw, Poland; k.wegrzynska@nil.gov.pl (K.W.); lidiaannabaraniak@gmail.com (L.B.); 3 Department of Basic Biomedical Science, Faculty of Pharmaceutical Sciences in Sosnowiec, Medical University of Silesia, 41-205 Sosnowiec, Poland; anna.wawrzyk@sum.edu.pl; 4 Faculty of Medicine, Collegium Medicum, WSB University, 41-300 Dabrowa Gornicza, Poland

**Keywords:** cancer patients, oncology hospital, epidemiological surveillance, multidrug-resistant *Klebsiella pneumoniae*, typing, Fourier transform infrared spectroscopy, IR Biotyper, PFGE

## Abstract

**Background/Objectives: ***Klebsiella pneumoniae* is one of the major causes of severe infections in cancer patients. The rapid and accurate typing of isolates is essential for tracking transmission routes and implementing infection control measures. The IR Biotyper, an automated system based on Fourier transform infrared (FT-IR) spectroscopy, enables fast cluster analysis with reduced cost and turnaround time. The aim of this paper was to compare typing results obtained by the IR Biotyper and pulsed-field gel electrophoresis (PFGE) for the epidemiological surveillance of *K. pneumoniae* in an oncology hospital. **Methods:** A total of 137 isolates collected between 2020 and 2023 from both colonization and infection were retrospectively analyzed using PFGE and the IR Biotyper. The discriminatory power of both methods and their concordance were assessed. **Results:** Both methods demonstrated high discriminatory power. PFGE classified the strains into 59 distinct types (96 including subtypes), while the IR Biotyper differentiated 70 FT-IR types. Concordance between the methods was moderate (adjusted Wallace coefficient: 0.515). PFGE type B was the most prevalent, comprising 43 isolates and subdivided into 16 subtypes. The most frequent FT-IR types were 16 (17 isolates), 10 (8 isolates), and 14 (5 isolates), all corresponding to PFGE type B with different subtypes. The IR Biotyper successfully distinguished isolates within the long-standing PFGE type B clone. **Conclusions:** The IR Biotyper demonstrated good discriminatory capacity and was able to differentiate isolates within a dominant PFGE clone, supporting its potential as a rapid tool for monitoring clonal spread in oncology settings. However, the moderate concordance with PFGE highlights that further studies are needed to optimize performance and confirm its role as a complementary method for routine hospital epidemiological surveillance.

## 1. Introduction

*Klebsiella pneumoniae* belongs to Enterobacterales and is a natural member of the gastrointestinal tract microbiome of humans and animals. It is an opportunistic pathogen that causes serious community-onset infections, such as necrotizing pneumonia or pyogenic liver abscesses. But first of all, *K. pneumoniae* is an important etiological factor of nosocomial infections, especially of the urinary tract, the respiratory tract, intra-abdominal and bloodstream [1,2]. Despite the wide range of available antibiotics, especially β-lactams, such as cephalosporins, monobactams and carbapenems, as well as aminoglycosides, fluoroquinolones, tetracyclines or co-trimoxazole, the treatment of *K. pneumoniae* infections is complicated due to acquired antimicrobial resistance (AMR). The most commonly detected acquired AMR mechanisms in this pathogen are the production of extended-spectrum β-lactamases (ESBLs), AmpC-like cephalosporinases and carbapenemases [3,4,5]. The spread of resistance to carbapenems is particularly dangerous because these drugs are mainly used as the last effective line of treatment for infections caused by multidrug-resistant (MDR) *K. pneumoniae* [6,7].

Infectious diseases are the main cause of morbidity and mortality among cancer patients due to immune deficiencies associated with malignancy itself and anticancer therapy [8,9,10]. Furthermore, infections can interfere with anticancer treatment, causing delays or even preventing its administration, which may affect short- and long-term outcomes of the underlying disease [8,11,12]. The main risk factor of *K. pneumoniae* infection is prior colonization of the gastrointestinal tract, and the disease is often caused by the colonizing strain [13]. The probability of colonization of cancer patients by MDR *K. pneumoniae* increases with repeated antibiotic treatment for infections and multiple hospitalizations in oncology facilities, where these strains can cause nosocomial outbreaks due to their ability to spread rapidly [14,15,16]. Considering that *K. pneumoniae* is a significant pathogen responsible for severe infections in cancer patients, the high prevalence and increasing number of MDR strains in oncology hospitals is a major clinical and epidemiological concern [8,17].

The rapid and accurate typing of *K. pneumoniae* isolates is a key tool for detecting potential transmission routes and identifying reservoirs of these pathogens as well as for infection prevention and control [18]. Pulsed-field gel electrophoresis (PFGE) has been widely used as the “gold standard” in bacterial typing for many years [19]. The success of this method was due to its applicability to all isolates (validity), capacity to distinguish unrelated isolates (discriminatory power), reproducibility both within and between laboratories (repeatability), and its cost-effectiveness and ease of implementation [19,20]. However, it should be noted that PFGE is a labor-intensive and time-consuming technique that requires qualified personnel. Furthermore, it is mostly used in retrospective epidemiological studies and therefore has no clinical application [21,22]. This remark also applies to highly selective whole genome sequencing (WGS), which is becoming the new gold standard in studies of isolate relationships [22]. Clinical microbiology laboratories require a method for the detection of pathogen cross-transmissions in real time, as well as early identification of nosocomial outbreaks, which can reduce patient hospitalization time and healthcare costs and above all prevent serious health complications caused by bacterial infections [23]. The IR Biotyper (Bruker Daltonics GmbH, Bremen, Germany), which uses Fourier transform infrared (FT-IR) spectroscopy, appears to be a system that meets all these expectations [24].

FT-IR spectroscopy is a spectrum-based technique that enables the quantitative determination of infrared radiation absorption by molecules present in a sample, such as lipids, nucleic acids, carbohydrates, lipopolysaccharides, and proteins. Since various particles absorb the radiation of different wavelengths, the analysis of a bacterial strain yields a unique pattern of characteristic spectral peaks. This pattern constitutes a specific “fingerprint” of the isolate and reflects its overall chemical composition. Since the polysaccharide region of the infrared spectrum exhibits the greatest spectral variability among strains of the same species, it is commonly employed for bacterial strain typing [21,22]. Due to the various advantages of FT-IR spectroscopy, including its high ability to distinguish between bacterial strains, ease of use, low cost and fast turnaround time, this technique has been implemented in the IR Biotyper, which enables fully automated spectral analysis and comparison using univariate, multivariate, and machine learning–based approaches, providing a robust tool for real-time epidemiological surveillance and infection control [21,22,24].

The objective of this paper was to conduct a comparative assessment of the IR Biotyper and PFGE for the epidemiological surveillance of *K. pneumoniae* in an oncology hospital. The analysis included isolates obtained from both colonization (carriage) and infection. This paper aimed to evaluate the discriminatory power and concordance of both methods in detecting clonal relatedness among isolates as well as to assess the applicability of the IR Biotyper as a rapid and reliable alternative to PFGE for routine hospital epidemiology and outbreak investigations in a high-risk clinical setting.

## 2. Materials and Methods

### 2.1. Clinical Isolates

The investigation included all *K. pneumoniae* strains collected between 2020 and 2023 from hospitalized patients at the Prof. F. Łukaszczyk Oncology Centre in Bydgoszcz. The isolates were obtained as part of routine microbiological testing for colonization and/or infection, and ethical approval was not required as the study formed part of standard laboratory diagnostics and infection control procedures. All strains from invasive infections (bloodstream) were stored and analyzed regardless of the presence of AMR mechanisms, whereas only AMR-positive isolates from other clinical specimens were retained and examined. The initial identification of bloodstream pathogens was performed using a BioFire Blood Culture Identification 2 (BCID 2) panel on the BioFire FilmArray System (BioFire Diagnostics, Salt Lake City, UT, USA), which was followed by the culture method. The species of isolates from all clinical specimens was confirmed by the matrix-assisted laser desorption/ionization time-of-flight mass spectrometry (IVD MALDI Biotyper Smart System; Microflex LT/SH Smart, Bruker Daltonik, Bremen, Germany). The strains were preserved in tryptone soy broth with 20% (*v*/*v*) glycerol (Graso Biotech, Starogard Gdański, Poland) and stored at −70 °C in a low-temperature freezer (NEXUS Slim EVO MD 810-VS; Angelantoni Life Science, Massa Martana, Italy), pending further analysis.

### 2.2. Susceptibility Testing and Phenotypic AMR Mechanisms Detection

Over the four-year period covered by this paper, the research methodology evolved. Antimicrobial susceptibility was assessed only for strains derived from infections using the Kirby–Bauer disc diffusion method [25] on Mueller-Hinton E agar (BioMérieux, Marcy-l’Étoile, France) or with the NMIC 417 panel with the automated nephelometric method of the BD Phoenix M50 system (Becton Dickinson, Sparks, MD, USA). Colistin susceptibility was determined by the broth microdilution method in accordance with the European Committee on Antimicrobial Susceptibility Testing (EUCAST) recommendations [26]. Quality control strains used in the assays included *Escherichia coli* ATCC 25922, *E. coli* ATCC 35218 and *K. pneumoniae* ATCC 700603, and the results were interpreted according to the EUCAST guidelines [26].

The presence of ESBL in isolates from infections previously tested by the disc diffusion method, as well as from colonization, was determined using the double disc synergy test (DDST) [27], since the NMIC 417 panel applied to the remaining strains also enables the detection of this type of β-lactamase. Carbapenemase screening breakpoints for isolates from infections were defined according to the EUCAST guidelines [26]. Colonization with carbapenemase-producing strains was evaluated with Brilliance CRE Agar (Thermo Scientific, Waltham, MA, USA). All suspected carbapenemase-producing isolates were subsequently subjected to the phenotypic detection of KPC, metallo-β-lactamase (MBL), and OXA-48 using the combined disk test with phenylboronic acid [28], DDST with EDTA [29], and the temocillin disc [30], respectively. Quality control strains used in the tests included *K. pneumoniae* ATCC 700603 (ESBL-positive control), *K. pneumoniae* NTCC 13438 (KPC-positive control), *K. pneumoniae* NTCC 13440 (MBL-positive control) and *K. pneumoniae* NTCC 13442 (OXA-48-positive control).

### 2.3. Molecular Identification of β-Lactamase

In bloodstream specimens analyzed by the BCID2 panel, carbapenemases and ESBLs from the CTX-M family were detected by nested multiplex polymerase chain reaction (PCR), while carbapenemase-producing strains identified phenotypically were routinely confirmed using the Xpert Carba-R assay (Cepheid, Sunnyvale, CA, USA) based on real-time PCR.

Prior to typing, all β-lactamases-producing isolates were comprehensively characterized by PCR assays for the presence of genes encoding ESBLs (*bla*_CTX-M-1_, *bla*_CTX-M-2_, *bla*_CTX-M-8_, *bla*_CTX-M-9_, *bla*_CTX-M-25_, *bla*_SHV-5_, *bla*_SHV-12_ and *bla*_TEM_), AmpC cephalosporinases (*bla*_CMY-2_ and *bla*_DHA_), and carbapenemases (*bla*_KPC_, *bla*_NDM_, *bla*_IMP_, *bla*_VIM_ and *bla*_OXA-48_), as previously described [31,32,33,34,35,36,37].

### 2.4. PFGE Typing

PFGE was performed according to the methodology described by Struelens et al. [38], using the *Xba*I restriction enzyme (Thermo Scientific, Waltham, MA, USA) and a CHEF DRIII PFGE system (Bio-Rad, Hercules, CA, USA). The resulting DNA profiles were visually analyzed according to the criteria proposed by Tenover et al. [39].

### 2.5. FT-IR Typing

FT-IR analysis was carried out with the IR Biotyper according to the manufacturer’s guidelines. All analyzed isolates were cultured on Columbia agar supplemented with 5% sheep blood (Oxoid, Wesel, Germany) and incubated at 35 ± 2 °C for 24 ± 0.5 h (Thermo Scientific, Waltham, MA, USA). Following the initial incubation, a further passage was performed on the same medium and incubated for an additional 24 ± 0.5 h. These 24 h cultures were used for the first series of determinations, comprising five technical replicates per isolate To obtain biological replication, an additional passage was undertaken. The resulting fresh 24 h cultures were used to prepare a new suspension and to carry out a second, independent series of determinations, again including five technical replicates. For each sample, 50 µL of 70% ethanol (Merck, Darmstadt, Germany) was combined with a small amount of bacterial biomass collected from the edge of the 24 h culture plate. After thorough vortexing, 50 µL of deionized water (Honeywell, Seelze, Germany) was added. Subsequently, 15 µL of the prepared suspension was dispensed onto a 96-well silicon target plate with five technical replicates prepared for each strain. In each series of determinations, 10 µL of infrared test standards IRTS 1 and IRTS 2 (Bruker Daltonics GmbH, Bremen, Germany) were applied in duplicate. The plates were left to dry completely at room temperature for approximately 30 min before analysis using the IR Biotyper. On the second day, the entire procedure was repeated using cultures obtained from biological replication, following the same suspension preparation and measurement protocol, resulting in a further five technical replicates. *K. pneumoniae* ATCC 700603 was included in each run as an internal quality control. Spectra were analyzed using the IR Biotyper v. 4.0.8 software (Bruker Daltonics GmbH, Bremen, Germany) with the manufacturer’s default settings. Samples that did not meet the quality criteria were excluded. For intergroup comparisons (dendrogram analysis), four representative spectra covering both replication types for each isolate were selected to reflect intra-strain variability and ensure consistent data presentation. The cutoff value (COV) for strain grouping was automatically determined by the IR Biotyper software by optimizing the product of the Simpson’s index of diversity (ID) and the mean cohesion (mC). For the 137 analyzed strains, the COV value was 0.205, and isolates clustered at or below this threshold were considered potentially clonally related. The same COV value was applied to the analysis of individual strains, allowing for a comparable assessment of clonal relatedness.

### 2.6. Typing Concordance

Simpson’s ID was determined for both methods. The types obtained by PFGE were compared with those obtained by IR Biotyper with an adjusted Rand (AR) index and adjusted Wallace (AW) coefficient with 95% confidence intervals (CI) using an online tool [40]. AR compares methods without considering any reference method to be superior and assesses the overall consistency between the two selection methods. In turn, AW compares the agreement of two typing methods considering one of them as the reference method. The AR and AW values can range from 0 to 1, where 0 indicates the expected random match and 1 indicates a perfect correlation between the two methods.

The discriminatory power of IR Biotyper was assessed using Simpson’s ID. This index estimates the probability that two unrelated strains randomly selected from a population will be assigned to different types. Values closer to 1 indicate that the strains are different, while values closer to 0 indicate that the strains are related.

## 3. Results

### 3.1. Data Collection

A total of 137 *K. pneumoniae* isolates collected from cancer patients hospitalized between 2020 and 2023 were analyzed. Of these, 69 were cultured from infections, including the urinary tract (*n* = 50) and bloodstream (*n* = 19), whereas 68 originated from colonization. The annual distribution of the tested strains is shown in Table 1.

Except for the 26 isolates recovered from 11 individuals (Table 2), all strains were obtained from different patients.

### 3.2. Phenotypic Characteristics and β-Lactamase Content

**Figure 1:**
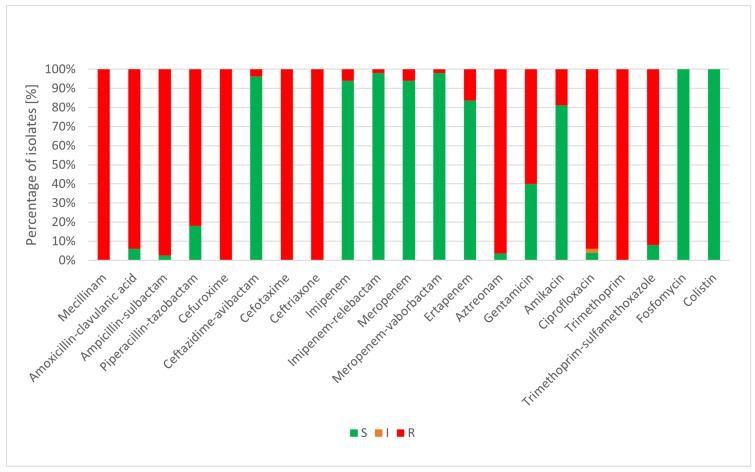
Susceptibility of *K. pneumoniae* isolated from urinary tract infections. Abbreviations: S—susceptible; I—susceptible, increased exposure; R—resistant.

**Figure 2:**
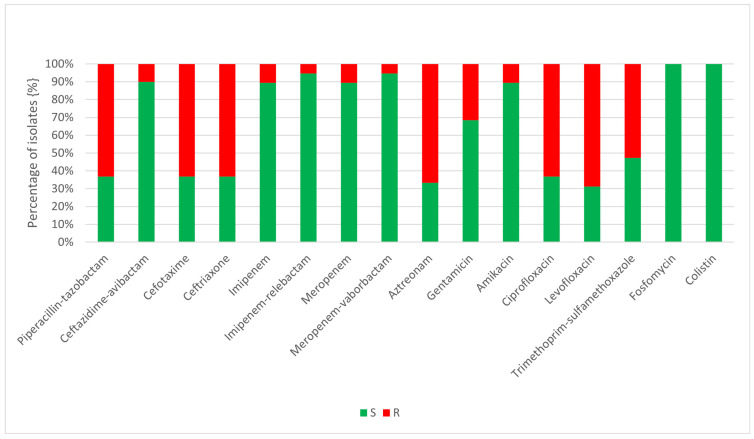
Susceptibility of *K. pneumoniae* isolated from bloodstream infections. Abbreviations: S—susceptible; R—resistant.

Only isolates obtained from infections were subjected to antimicrobial susceptibility testing. The selection of antibiotics was performed in accordance with national guidelines [41] and reflected the type of infection from which the strain originated. The susceptibility results are presented in Figure 1 and Figure 2.

All urinary tract infection isolates were resistant to mecillinam, cefuroxime, cefotaxime, ceftriaxone, and trimethoprim. Moreover, most strains exhibited resistance to penicillin–inhibitor combinations, aztreonam, ciprofloxacin, and trimethoprim with sulfamethoxazole. Ertapenem and amikacin retained activity against a substantial proportion of isolates. In turn, ceftazidime with avibactam, imipenem, imipenem with relebactam, meropenem, meropenem with vaborbactam, fosfomycin, and colistin were the most active drugs in vitro, but resistance to some of these was occasionally observed.

More than half of the bloodstream infection isolates exhibited resistance to piperacillin with tazobactam, cefotaxime, ceftriaxone, aztreonam, ciprofloxacin, levofloxacin, and trimethoprim with sulfamethoxazole. The majority of strains remained susceptible to ceftazidime with avibactam, imipenem, imipenem with relebactam, meropenem, meropenem with vaborbactam, and amikacin. All isolates were susceptible to fosfomycin and colistin.

Except for the seven isolates obtained from bloodstream infections (*n* = 3, *n* = 2, *n* = 1 and *n* = 1 in 2020, 2021, 2022 and 2023, respectively), all remaining strains (*n* = 130) were classified as putative ESBL producers based on positive test results. In all ESBL-positive strains, enzymes belonging to the CTX-M family were identified using specific PCR assays. The majority carried CTX-M-1-like enzymes (*n* = 128), whereas CTX-M-9-like enzymes were detected only in two isolates: one recovered in 2020 and one in 2021. Ninety-eight strains (72%) harbored TEM-1-like β-lactamases. Carbapenemase production was detected in 18 isolates, predominantly NDM (*n* = 9; two isolates in each of the first three years and three isolates in 2023), followed by KPC (*n* = 6) and OXA-48-like enzymes (*n* = 3), with all KPC- or OXA-48-like-producing strains isolated in 2023. All NDM-producing *K. pneumoniae*, except for two isolates, were obtained from colonizations, one strain being from the urinary tract and one from bloodstream infection. OXA-48-producing isolates were all from colonization, while KPC-producing isolates were recovered from both carriage (*n* = 3) and infections (*n* = 2 from urinary tract and *n* = 1 from bloodstream).

### 3.3. PFGE Typing

All 137 isolates were analyzed by PFGE, and the results are shown in Table 3.

A total of 59 distinct PFGE types were identified among the analyzed strains. Eighteen of these (A, B, F, G, N, O, W, X, Z, AA, AC, AE, AG, AJ, AN, AT, AY and AAD) included more than one isolate and were further subdivided into subtypes. Type B was the most prevalent, comprising 43 strains collected throughout the study period and split into 16 subtypes. This type included strains originating from both colonization and infection. Among the type B subtypes, B12 was the most frequent (*n* = 9), followed by B1 (*n* = 8), B8 (*n* = 5), B9 (*n* = 5), B2 (*n* = 4), and B16 (*n* = 2).

### 3.4. FT-IR Typing

All 137 isolates were analyzed by the IR Biotyper, and the results are presented in Table 4.

A total of 70 FT-IR types were identified among the analyzed strains, 22 of which (1–22) comprised multiple isolates. Type 16 was the most prevalent with 17 strains collected throughout the study period. This type included seven isolates from colonization (*n* = 4 and *n* = 3 in 2020 and 2021, respectively) and ten isolates from infection (*n* = 2, *n* = 6, *n* = 1, and *n* = 1 in 2020, 2021, 2022, and 2023, respectively).

### 3.5. Typing Concordance

PFGE and the IR Biotyper showed high discriminatory power with Simpson’s ID values of 0.989 (95% CI: 0.982–0.995) and 0.973 (95% CI: 0.961–0.986), respectively. The overall agreement between the two typing methods was reflected by an AR index of 0.304. The directional concordance assessed using the AW coefficient was 0.515 (95% CI: 0.337–0.694) from PFGE to the IR Biotyper and 0.216 (95% CI: 0.115–0.317) from the IR Biotyper to PFGE

PFGE typing differentiated the 137 *K. pneumoniae* isolates into multiple subtypes, some of which corresponded closely to single FT-IR types, whereas others were subdivided across several FT-IR types. A comparison of PFGE typing results for the most prevalent type B with the results obtained using the IR Biotyper is presented in Table 5.

Overall, strains belonging to PFGE type B were assigned 11 different FT-IR types. Notably, the predominant PFGE subtype B12, comprising nine isolates, mostly corresponded to FT-IR type 16 (7/9 isolates) with the remaining two strains classified as types 57 and 59. Importantly, these two isolates were recovered from different clinical specimens (urine and blood), from different patients and hospital wards, and were collected at different time points over a two-year period. PFGE subtype B1 included eight isolates, six of which were assigned to FT-IR type 10, while the other two were assigned to types 14 and 18. PFGE subtypes B8 and B9, each comprising five isolates, were subdivided into three and four FT-IR types, respectively, reflecting intra-subtype heterogeneity. Subtype B8 included isolates assigned to types 10 (*n* = 1), 14 (*n* = 2), and 16 (*n* = 2), whereas subtype B9 comprised types 14 (*n* = 1), 16 (*n* = 2), 19 (*n* = 1), and 32 (*n* = 1). Several PFGE subtypes such as B2 (*n* = 4) and B16 (*n* = 2) were homogeneous with all isolates assigned to a single FT-IR type (16 and 18, respectively).

Interestingly, the remaining PFGE types, which grouped more than one isolate, mostly classified the strains into a single FT-IR type (Table S1), demonstrating concordance between methods for less diverse groups. Among the five strains of type A (divided into four subtypes), four were designated as type 22. Of the four strains of type N (two subtypes), three were assigned to type 1. Both strains of type O were classified as type 6. Among the three strains of type W (two subtypes), two belonged to type 2. Four strains belonging to type X (three subtypes) were classified into two types, 5 and 17. All isolates of type AG (three subtypes) were assigned to type 4. Two strains each from types AJ (two subtypes) and AT (one subtype) were classified as types 7 and 9, respectively.

**Figure 3:**
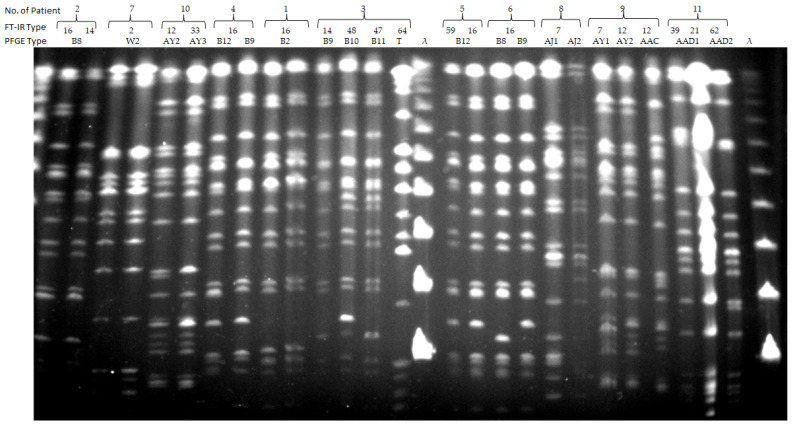
PFGE profiles of the *K. pneumoniae* isolates from 11 patients; compilation of PFGE results and corresponding IR Biotyper results. The lane designated λ contained the molecular size marker (λ ladder DNA: 48.5–1018 kb; New England Biolabs, Ipswich, MA, USA).

**Figure 4:**
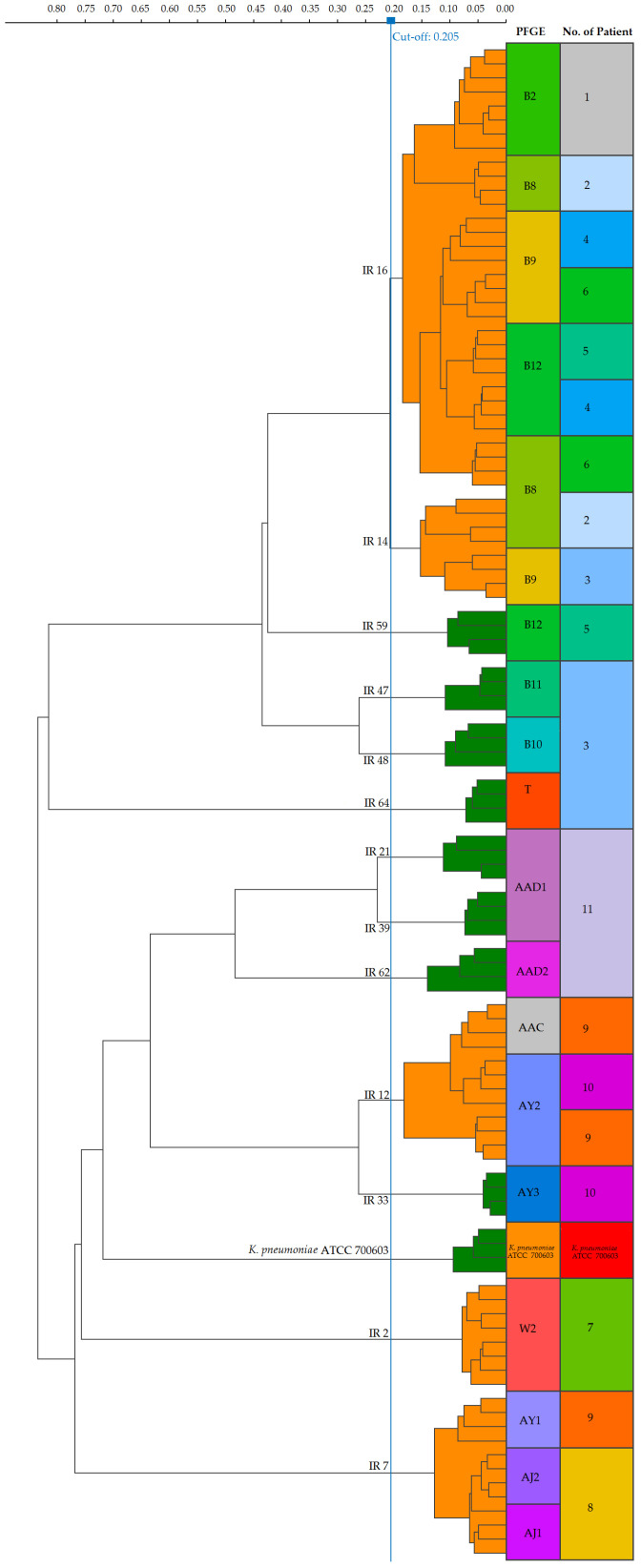
Dendrogram obtained by IR Biotyper for the *K. pneumoniae* isolates from 11 patients; compilation of FT-IR types and corresponding PFGE types. *K. pneumoniae* ATCC 700603 strain was used as the reference material. The vertical dashed line indicates the used cutoff value (0.205). Resulting FT-IR types are shown in orange and green.

A separate comparative analysis of the relatedness of *K. pneumoniae* isolates obtained from the same individual was also performed for all 11 patients, and the results are shown in Figure 3 and Figure 4.

At the patient level, multiple isolates from the same individual allowed for an assessment of strain persistence, the acquisition of new strains, and the concordance between PFGE and FT-IR typing. Two isolates obtained from patient no. 1 were indistinguishable by PFGE (B2) and shared the same FT-IR type (16), likely indicating that the colonizing strain caused the subsequent infection, with full agreement between methods. Patient no. 2 had isolates obtained over a two-year period: the first from carriage and the second from a urinary tract infection. PFGE showed that both isolates belonged to the same type (B8), indicating the same strain, while FT-IR assigned them to different but closely related types, 16 and 14, reflecting minor phenotypic variation over time. Four isolates from patient no. 3 included three belonging to a single PFGE type B (B9, B10, and B11) and one unrelated strain (T). In contrast, FT-IR analysis classified these isolates into four distinct types: 14, 48, 47, and 64, demonstrating the limited concordance between methods. Two isolates belonging to PFGE type B (B12 and B9) were cultured from patient no. 4 and were assigned to a single FT-IR type (16), showing good agreement. Two strains indistinguishable by PFGE (B12) from patient no. 5 exhibited different FT-IR types, 59 and 16, indicating limited concordance. Patient no. 6 had two isolates belonging to PFGE type B (B8 and B9) classified as a single FT-IR type (16), reflecting partial agreement despite PFGE differentiation. Patient no. 7 was characterized by two isolates that were identical by both typing methods (W2 and 2, respectively). Two PFGE AJ isolates (AJ1 and AJ2) from patient no. 8 corresponded to FT-IR type 7, showing full concordance. Of three strains from patient no. 9, two belonged to PFGE type AY (AY1 and AY2), while the third was unrelated (AAC). FT-IR typing assigned them to types 7 and 12 with the two PFGE-different isolates grouped under FT-IR type 12. Two isolates from patient no. 10, both of the PFGE AY types (AY2 and AY3) were classified as FT-IR types 12 and 13, showing minor discordance. Finally, three isolates obtained from patient no. 11 belonged to a single PFGE type (two AAD1, one AAD2) and had distinct FT-IR types 39, 21, and 62, reflecting limited concordance between methods.

In the dendrogram, the 26 isolates obtained from 11 patients were assigned to 13 FT-IR types. Clustered isolates are indicated in orange and comprised five FT-IR types (16, 14, 12, 2 and 7), whereas individually occurring isolates are shown in green and represented eight FT-IR types (59, 47, 48, 64, 21, 39, 62 and 33). PFGE analysis differentiated these last isolates into 15 profiles, including nine unique types.

## 4. Discussion

Although PFGE and WGS are considered the “gold standards” for the molecular/genomic typing of isolates from patients and their environment during both the investigation and control of nosocomial outbreaks, their previously mentioned limitations make them unsuitable for routine large-scale bacterial typing in public health departments [19,42]. Therefore, alternative typing approaches are being explored to enable clinical microbiology laboratories to rapidly and efficiently detect potentially linked strains. In this paper, we compared the relatedness of *K. pneumoniae* isolates assessed using PFGE as the reference method and the IR Biotyper in order to evaluate the usefulness of this new automated typing tool for monitoring pathogen spread and outbreak investigations in an oncology hospital.

Comparative investigations require the use of a well-characterized study group and experimental conditions that closely reflect those under which the new typing method will ultimately be applied in clinical practice. The tested *K. pneumoniae* population comprised all ESBL-positive isolates from colonization and urinary tract infection and both ESBL-positive and ESBL-negative isolates from bloodstream infection obtained from hospitalized cancer patients between 2020 and 2023. The number identified annually was comparable, remaining stable at around 30–40 per year since 2019, as previously reported [11,43]. They consisted of almost equal amounts of isolates from colonization and infection. All *K. pneumoniae* were comprehensively characterized for β-lactamase production, and strains isolated from infections were additionally tested for antimicrobial susceptibility. The vast majority of isolates (95%) harbored ESBLs of the CTX-M family, predominantly CTX-M-1-like (99%), while CTX-M-9-like enzymes were detected in two isolates. A similar epidemiological situation was observed for *K. pneumoniae* in 2019, with all ESBL-producing isolates harboring β-lactamases of the CTX-M family, which were identified as CTX-M-15 for strains from infections. Interestingly, in 2019, enzymes from the CTX-M-9 family, including CTX-M-27 and CTX-M-65, were detected in two other species, *E. coli* and *Enterobacter cloacae*, respectively [11,43]. This suggests that these ESBLs may have been acquired by *K. pneumoniae* via horizontal gene transfer (e.g., conjugation). As in 2019, NDM-producing *K. pneumoniae* was detected sporadically each year in this paper, with earlier isolates originating exclusively from colonization, while in 2022 and 2023, single strains were recovered from urinary tract and bloodstream infections, respectively [43]. It is concerning that in 2023, nine isolates carrying other carbapenemases, KPC and OXA-48, were identified, as these enzymes had not been detected in previous years. Of these, three KPC-producing isolates originated from infections, two from the urinary tract and one from the bloodstream. As the vast majority of the study group harbored ESBLs, nearly all were MDR and remained fully susceptible only to meropenem combined with vaborbactam, fosfomycin, and colistin.

All *K. pneumoniae* isolates were typed using both PFGE and IR Biotyper. The reference method classified the strains into 59 distinct types (total 96, including subtypes), whereas the evaluated tool differentiated 70 FT-IR types. PFGE type B was the most prevalent and was detected in all of the analyzed years. Notably, this type had already been observed in 2019 in isolates from surgical wound and urinary tract infections, where it was classified as type C [11]. PFGE type B included 43 isolates from both colonization and infections and was subdivided into 16 subtypes. Subtype B12 was the most prevalent, comprising nine isolates, which was followed closely by B1 with eight isolates. In contrast, the most frequent FT-IR type was 16, which grouped 17 isolates, which was followed by type 10 with eight isolates. Interestingly, FT-IR 16 showed a distinct temporal distribution, being limited to colonization strains during the first two years of the study, but remaining among infection isolates throughout the study period. The FT-IR 10 type was detected in isolates from colonization only in 2020, which was also the year with the highest number of isolates from infections. In the following two years, only one strain from infections was identified per year, and in 2023, this type was no longer observed. Importantly, all isolates assigned to FT-IR types 16 and 10, as well as type 14, which included five strains, were classified by PFGE as type B (with different subtypes).

Both PFGE and the IR Biotyper demonstrated high discriminatory power, as reflected by Simpson’s ID values of 0.989 (95% CI: 0.982–0.995) and 0.973 (95% CI: 0.961–0.986), respectively. However, moderate concordance between the two methods was observed, as indicated by an AR index of 0.304, reflecting differences in strain clustering and highlighting that further studies are required to optimize the IR Biotyper’s performance and establish its role as a complementary tool for routine hospital epidemiological surveillance. Directional concordance analysis revealed an asymmetrical relationship between the two typing methods. The probability that strains clustered by PFGE were also assigned to the same type by the IR Biotyper was 51.5% (AW = 0.515; 95% CI: 0.337–0.694), whereas concordance in the reverse direction was lower at 21.6% (AW = 0.216; 95% CI: 0.115–0.317). A similar discriminatory performance of the IR Biotyper for *K. pneumoniae* has been reported by Wang-Wang et al., who observed an AW of approximately 0.52 and comparable Simpson’s ID and AR values using WGS as the reference method [44]. By contrast, other studies comparing the IR Biotyper with PFGE, multi-locus sequence typing, or WGS reported substantially higher concordance, with AR indices reaching 0.958 and AW values ranging from 0.934 to 0.983, highlighting that observed agreement is strongly influenced by the reference method employed [22,45]. These differences may also reflect the number of analyzed isolates, the period of collection, and the diversity of participating centers. As a relatively novel machine learning-based approach, the IR Biotyper requires further data collection to fully assess its performance. Nevertheless, its moderate concordance with PFGE indicates that it can serve as a rapid and efficient screening method for potential clusters, facilitating timely infection control decisions and optimizing resource allocation in routine hospital surveillance, while confirmation with reference methods remains advisable.

It should be noted that PFGE relies on the action of a specific restriction enzyme that cleaves DNA at defined recognition sites. Consequently, genetic changes occurring outside these regions remain undetected, and isolates may appear indistinguishable by PFGE despite minor genomic and/or phenotypic variation. In contrast, FT-IR captures broader phenotypic characteristics, including differences in cell surface composition and metabolic profiles, which may reflect the ongoing adaptation or microevolution of strains. FT-IR spectra are strongly influenced by the biochemical composition of the bacterial cell envelope, particularly capsular polysaccharides, lipopolysaccharides, and other surface associated macromolecules. The expression and structural modification of these components are dynamic and may be modulated by environmental factors such as nutrient availability, oxidative stress, temperature, osmotic pressure, and exposure to antimicrobial agents. Under in vivo conditions, especially in immunocompromised patients, additional host-related factors including immune pressure, inflammation, and multiple antibiotic therapy may further influence surface bacterial polysaccharide expression. As a result, isolates belonging to the same PFGE type may exhibit measurable spectral differences attributable to adaptive phenotypic variation rather than true genetic divergence. This may explain why PFGE identical isolates are assigned to different FT-IR types and further underscores the complementary nature of these approaches. While PFGE primarily assesses genetic relatedness, FT-IR enables the detection of phenotypic heterogeneity between strains, providing additional epidemiological insight. In clinical settings, particularly among cancer patients, capturing such intra-strain variability may be crucial for understanding persistence, transmission dynamics, and potential evolutionary trajectories over time.

### Limitations

This paper has several limitations. First, the study was conducted at a single center and the sample size was relatively small, which may limit the generalizability of the findings to other hospitals or regions. Second, it was conducted using stored isolates and the IR Biotyper was not evaluated in real time during an outbreak. Third, while all bloodstream isolates were included regardless of their AMR mechanisms, only AMR-positive strains from other clinical specimens were analyzed. This may lead to an underrepresentation of susceptible isolates from non-invasive infections and could influence the observed diversity. Fourth, multiple isolates from the same patient were included to capture both the persistence of strains over time and the potential acquisition of unrelated clones. Although restricting the analysis to a single isolate per patient could reduce intra-patient clustering, any selection criterion (e.g., first isolate, last isolate, or isolate from a particular specimen) would be arbitrary and might introduce bias. Repeated isolates account for 26 of 137 strains (~19% of the total) and do not dominate the clustering results. Including all isolates provides a more comprehensive reflection of strain dynamics, particularly in high-risk populations such as cancer patients, and allows for a more accurate evaluation of the discriminatory ability and consistency of typing methods. Fifth, this paper focused solely on clinical isolates and did not include environmental samples, such as surface or equipment swabs. This limits the ability to identify potential hospital reservoirs and fully understand the transmission routes of MDR clones in oncology patients.

## 5. Conclusions

In conclusion, the IR Biotyper demonstrated high discriminatory power in differentiating *K. pneumoniae* isolates from cancer patients, although concordance with PFGE was moderate. It was able to distinguish strains of PFGE type B, which have been present in the hospital for at least five years, highlighting its potential for monitoring pathogen spread in an oncology setting. These findings support the continued collection of data to further evaluate the IR Biotyper as a complementary tool for epidemiological surveillance.

## Figures and Tables

**Table 1:** Number of isolates per year by clinical specimen.

Year	Carriage	Urinary Tract	Bloodstream	Total No.
2020	20	10	4	34
2021	20	12	7	39
2022	10	20	3	33
2023	18	8	5	31

**Table 2:** Collection data of multiple isolates from the same patients.

No. of Patient	Total No. of Isolates	Date of Isolation from Carriage	Date of Isolation from Urinary Tract	Date of Isolation from Bloodstream
1	2	11 February 2020		21 February 2020
2	2	6 August 2020	19 March 2022	
3	4		14 December 2020, 9 March 2021	14 February 2021, 9 March 2021
4	2		19 May 2021	14 September 2022
5	2		7 June 2021	7 June 2021
6	2		11 August 2021	8 August 2021
7	2		15 November 2021	15 November 2021
8	2		19 May 2022, 31 May 2022	
9	3	30 May 2023	25 May 2023	10 June 2023
10	2	30 May 2023	10 October 2023	
11	3		18 June 2023, 28 June 2023	28 June 2023

**Table 3:** Relatedness of *K. pneumoniae* isolates using PFGE typing.

Total No. of Isolates	PFGE		No. of Isolates from							
			Carriage				Infection ^1^			
	Type	Subtype	2020	2021	2022	2023	2020	2021	2022	2023
5	A	A1	1		1					
		A2				1				
		A3				1				
		A4								1b
43	B	B1	2	1			3u	1u	1u	
		B2	3				1b			
		B3	1							
		B4					1u			
		B5	1							
		B6	1							
		B7	1							
		B8	1				1u	1b	2u	
		B9					1u	2u, 1b	1b	
		B10						1b		
		B11						1u		
		B12		2				4u, 1b		2u
		B13		1						
		B14						1u		
		B15		1						
		B16			1	1				
1	C		1							
1	D		1							
1	E						1u			
2	F	F1	1							
		F2					1u			
2	G		2							
1	H		1							
1	I						1u			
1	J		1							
1	K						1b			
1	L						1u			
1	M		1							
4	N	N1					1b			
		N2				3				
2	O		1	1						
1	P						1b			
1	R			1						
1	S			1						
1	T							1b		
1	U			1						
3	W	W1		1						
		W2						1u, 1b		
5	X	X1		1		1				
		X2			1					
		X3			1				1u	
1	Y			1						
2	Z			2						
3	AA	AA1		1						
		AA2						1u		
		AA3		1						
1	AB			1						
2	AC			2						
1	AD							1u		
5	AE	AE1		1						
		AE2			1					
		AE3							2u	
		AE4			1					
1	AF							1b		
3	AG	AG1							1u	
		AG2							1u	
		AG3							1u	
1	AH								1u	
1	AI								1b	
2	AJ	AJ1							1u	
		AJ2							1u	
1	AK				1					
1	AM								1u	
2	AN	AN1			1					
		AN2							1u	
1	AO								1u	
1	AP				1					
1	AR								1b	
1	AS								1u	
2	AT								2u	
1	AU				1					
1	AW								1u	
1	AX								1u	
4	AY	AY1								1u
		AY2				2				
		AY3								1u
1	AZ									1u
1	AAA					1				
1	AAB									1b
1	AAC									1b
4	AAD	AAD1				1				2u
		AAD2								1b
1	AAE					1				
1	AAF					1				
1	AAG									1b
1	AAH					1				
1	AAI					1				
1	AAJ									1u
1	AAK					1				
1	AAL					1				
1	AAM					1				
137	59	48	20	20	10	18	14	19	23	13

^1^ u—urinary tract infection isolate; b—bloodstream infection isolate.

**Table 4:** Relatedness of *K. pneumoniae* isolates using the IR Biotyper.

Total No. of Isolates	FT-IR Type	No. of Isolates from							
		Carriage				Infections ^1^			
		2020	2021	2022	2023	2020	2021	2022	2023
3	1				3				
4	2	2					1u, 1b		
2	3		1	1					
3	4							3u	
2	5		1		1				
2	6	1	1						
3	7							2u	1u
2	8				1				1u
5	9	1			1			3u	
8	10	3				3u	1u	1u	
2	11	1	1						
4	12				3				1b
2	13	1			1				
5	14	1				2u		2u	
2	15	1				1u			
17	16	4	3			1u, 1b	5u, 1b	1b	1u
3	17			2		1u			
6	18	1	1	1	1			1u, 1b	
3	19	1					2		
3	20			1		1u		1u	
3	21				2				1u
5	22	1		1	1			1u	1b
1	23							1u	
1	24						1u		
1	25			1					
1	26						1u		
1	27							1u	
1	28				1				
1	29		1						
1	30								1u
1	31	1							
1	32						1b		
1	33								1u
1	34		1						
1	35		1						
1	36		1						
1	37		1						
1	38		1						
1	39								1u
1	40		1						
1	41					1b			
1	42		1						
1	43								1b
1	44	1							
1	45							1u	
1	46			1					
1	47						1u		
1	48						1b		
1	49				1				
1	50								1b
1	51							1b	
1	52		1						
1	53			1					
1	54				1				
1	55					1b			
1	56			1					
1	57								1u
1	58		1						
1	59						1b		
1	60							1u	
1	61					1u			
1	62								1b
1	63				1				
1	64						1b		
1	65						1b		
1	66					1b			
1	67							1u	
1	68		1						
1	69							1u	
1	70		1						
137	70	20	20	10	18	14	19	23	13

^1^ u—urinary tract infection isolate; b—bloodstream infection isolate.

**Table 5:** Compilation of PFGE B subtypes and corresponding FT-IR types.

No. of Isolates												
	FT-IR Type	10	14	16	18	19	32	36	47	48	57	59
PFGE B Subtype												
**B1**		6	1		1							
**B2**				4								
**B3**			1									
**B4**				1								
**B5**					1							
**B6**		1										
**B7**						1						
**B8**		1	2	2								
**B9**			1	2		1	1					
**B10**										1		
**B11**									1			
**B12**				7							1	1
**B13**								1				
**B14**						1						
**B15**				1								
**B16**					2							

## Data Availability

All of the original results presented in this paper are included within the paper. Additional information can be obtained from the corresponding authors.

## Supplementary Materials

The following supporting information can be downloaded at https://www.mdpi.com/article/10.3390/jcm15062301/s1, Table S1: Compilation of PFGE types grouping multiple isolates with corresponding FT-IR types.

## Abbreviations

The following abbreviations are used in this manuscript:

AMR	antimicrobial resistance
ESBL	extended-spectrum β-lactamase
MDR	multidrug-resistant
PFGE	pulsed-field gel electrophoresis
WGS	whole genome sequencing
FT-IR	Fourier transform infrared
S	susceptible
I	susceptible, increased exposure
R	resistant
Simpson’s ID	Simpson’s index
CI	confidence intervals
AR index	adjusted Rand index
AW coefficient	adjusted Wallace coefficient
EUCAST	European Committee on Antimicrobial Susceptibility Testing
BCID 2 panel	Blood Culture Identification 2 panel
DDST	double disc synergy test
MBL	metallo-β-lactamase
