# Supplementary material for: Comparative Assessment of the IR Biotyper and Pulsed-Field Gel Electrophoresis (PFGE) for Epidemiological Surveillance of Klebsiella pneumoniae in an Oncology Hospital
Source: J Clin Med. 2026 Mar 17;15(6):2301. doi: 10.3390/jcm15062301 (PMC13026370; doi:10.3390/jcm15062301)
Supplement: Supplementary file 1 [file jcm-15-02301-s001.zip › jcm-4164467-supplementary.pdf]

Table S1. Compilation of PFGE types grouping multiple isolates with corresponding FT-IR types.

FT-IR Type / PFGE B Subtype	1	2	3	4	5	6	7	9	11	12	13	15	17	18	20	21	22	24	27	29	33	34	35	37	38	39	40	56	58	62	66	67
A1																	2															
A2																	1															
A3											1																					
A4																	1															
F1												1																				
F2															1																	
G								1			1																					
N1																															1	
N2	3																															
O						2																										
W1																				1												
W2		2																														
X1					2																											
X2													1																			
X3													1						1													
Z									1																				1			
AA1																									1							
AA2																		1														
AA3																											1					
AC																						1	1									
AE1																								1								
AE2															1																	
AE3														1	1																	
AE4																												1				
AG1				1																												
AG2				1																												

AG3				1																												
AJ1							1																									
AJ2							1																									
AN1			1																													
AN2																																1
AT								2																								
AY1							1																									
AY2										2																						
AY3																					1											
AAD1																2										1						
AAD2																														1		
